# Biochemical and cytological interactions between callose synthase and microtubules in the tobacco pollen tube

**DOI:** 10.1007/s00299-022-02860-3

**Published:** 2022-03-18

**Authors:** Luigi Parrotta, Claudia Faleri, Cecilia Del Casino, Lavinia Mareri, Iris Aloisi, Gea Guerriero, Jean-Francois Hausman, Stefano Del Duca, Giampiero Cai

**Affiliations:** 1 Department of Biological, Geological and Environmental Sciences, University of Bologna, Via Irnerio 42, 40126 Bologna, Italy; 2 Interdepartmental Centre for Agri-Food Industrial Research, University of Bologna, Via Quinto Bucci 336, 47521 Cesena, Italy; 3 Department of Life Sciences, University of Siena, Via P.A. Mattioli 4, 53100 Siena, Italy; 4 Research and Innovation Department, Luxembourg Institute of Science and Technology, 5 Avenue des Hauts-Fourneaux, 4362 Esch/Alzette, Luxembourg

**Keywords:** Callose synthase, Microtubule, Pollen tube, Actin filaments, Callose

## Abstract

**Key message:**

The article concerns the association between callose synthase and cytoskeleton by biochemical and ultrastructural analyses in the pollen tube. Results confirmed this association and immunogold labeling showed a colocalization.

**Abstract:**

Callose is a cell wall polysaccharide involved in fundamental biological processes, from plant development to the response to abiotic and biotic stress. To gain insight into the deposition pattern of callose, it is important to know how the enzyme callose synthase is regulated through the interaction with the vesicle-cytoskeletal system. Actin filaments likely determine the long-range distribution of callose synthase through transport vesicles but the spatial/biochemical relationships between callose synthase and microtubules are poorly understood, although experimental evidence supports the association between callose synthase and tubulin. In this manuscript, we further investigated the association between callose synthase and microtubules through biochemical and ultrastructural analyses in the pollen tube model system, where callose is an essential component of the cell wall. Results by native 2-D electrophoresis, isolation of callose synthase complex and far-western blot confirmed that callose synthase is associated with tubulin and can therefore interface with cortical microtubules. In contrast, actin and sucrose synthase were not permanently associated with callose synthase. Immunogold labeling showed colocalization between the enzyme and microtubules, occasionally mediated by vesicles. Overall, the data indicate that pollen tube callose synthase exerts its activity in cooperation with the microtubular cytoskeleton.

**Supplementary Information:**

The online version contains supplementary material available at 10.1007/s00299-022-02860-3.

## Introduction

In plant cells, callose is a β-1,3-linked glucose polymer with some 1,6-branches (Chen and Kim 2009; Zaveska and Honys 2017), which accumulates under abiotic and biotic stresses (Albrecht and Mustroph 2003; Ellinger and Voigt 2014) as well as during specific developmental stages such as control of plasmodesmata trafficking (Xie et al. 2011) and reproduction (both sexual and apomictic) (Xiaoyun et al. 2005). Therefore, callose is a multifunctional polymer involved in several aspects of plant growth and development (Luna et al. 2011).

Callose is synthesized at the plasma membrane level by the enzyme callose synthase (CalS), which is activated by the lipophilic environment, and directly released into the cell wall (Brownfield et al. 2008). The enzyme is not exclusively associated with the plasma membrane but also with other subcellular compartments, which likely represent intermediates in the delivery process (Turner et al. 1998; Böhlenius et al. 2010). CalS is hypothesized to be inserted inactive into the plasma membrane, and subsequently converted by proteolytic events into the active form (Brownfield et al. 2008). Several genes are responsible for the expression of CalS isoforms. For example, the *Arabidopsis thaliana* gene *AtGSL5* is important during callose synthesis in flowers (Shi et al. 2015) as well as for papillary and wound-induced callose synthesis (Voigt 2014); while CalS encoded by the *AtGSL8* gene is critical for cell division and tissue patterning (Toller et al. 2008). The *AtGSL8* gene is also involved in the auxin feedback loop that regulates callose levels in plasmodesmata so as to locally downregulate symplast permeability during the tropic hypocotyl response in *Arabidopsis* (Han et al. 2014). Evidence indicates that the *AtGSL7* gene encodes for a CalS critical for phloem development and function (Barratt et al. 2011). The presence of related CalS genes suggests that they may perform related but overlapping functions, such as the *AtGSL1* and *AtGSL5* genes in *Arabidopsis*, which play redundant roles in sporophyte and pollen development (Xie et al. 2012).

In the pollen tube of angiosperms, callose is deposited as a tubular sheath surrounding the pollen tube flanks. Callose is usually absent at the apex and subapex of pollen tubes, and it is a common feature that incidental presence in those regions is associated with cessation of growth (Geitmann and Steer 2006; Mandrone et al. 2019; Aloisi et al. 2020). The tubular sheath of callose enhances the tensile strength of the cell wall. Indeed, callose deposition counteracts turgor pressure within pollen tubes so that the latter can be exerted only at the apex. Here, the absence of callose allows expansion of the methylated pectin-enriched soft hemispheric domain supporting the finger-like shape of pollen tubes, a prerequisite for growth through the stigma and style. Together with vesicular secretion, this process drives pollen tube growth (Chebli et al. 2012). During pollen maturation and pollen tube germination, callose is likely synthesized by the *AtGSL5* gene in *Arabidopsis* (Shi et al. 2015). In *Nicotiana alata*, the *NaGSL1* gene encodes a CalS with a molecular weight of 220 kD (Brownfield et al. 2007) that is produced after pollen germination. The trafficking of CalS to the plasma membrane probably proceeds through the endoplasmic reticulum, Golgi bodies, and intracellular vesicles inserted into the plasma membrane (Brownfield et al. 2008).

CalS trafficking, insertion, and activation are dependent on the cytoskeleton, but their exact molecular interaction is unclear. In the pollen tube, both actin filaments and endomembranes are critical for the distribution of CalS, which is likely transported in Golgi bodies and vesicles that move along actin filaments (Cai et al. 2011). As further support, in shorter pollen tubes CalS is found primarily in the Golgi and endoplasmic reticulum, whereas it accumulates within vesicles in longer pollen tubes (Brownfield et al. 2008). Although this does not indicate the direct involvement of actin filaments, it does suggest that CalS travels through the endomembrane system, which is known to be associated with actin filaments. The association between CalS and actin filaments may not be limited to vesicular transport; in the secondary vascular tissues of angiosperms and gymnosperms, the pores of sieve elements and sieve cells are surrounded by myosin and actin in addition to callose (Chaffey and Barlow 2002).

The association between CalS and microtubules is even less known (Chebli et al. 2021). In *Riella helicophylla*, increased callose deposition is linked to the removal of microtubules or altered microtubule dynamics, suggesting that CalS insertion or activation is strictly dependent on proper microtubule organization (Scherp et al. 2002). Indirect evidence for the association between CalS and microtubules is found in cotton fibers where sucrose synthase accumulates between the cortical microtubules and plasma membrane and in a proximal exoplasmic zone about 0.2 µm thick also characterized by callose deposition (Salnikov et al. 2003). The interaction between microtubule depolymerization and callose is also supported by caffeine-mediated inhibition of callose deposition in cells not yet undergoing mitotic division (Yasuhara 2005). All this shows that both CalS insertion and activation are related to microtubule dynamics but does not reveal their molecular connection. In *Arabidopsis* and tobacco suspension cells, destabilization of microtubules by oryzalin negatively affects callose synthesis, with tubulin and CalS forming a complex in the plasma membrane (Aidemark et al. 2009). In pollen tubes, CalS is associated with the plasma membrane in the apex and distal regions, as well as around callose plugs. The use of inhibitors indicated that actin filaments and endomembranes are critical for the apical distribution of CalS, whereas microtubules are important in positioning CalS in distal regions and around callose plugs; native electrophoresis indicated that CalS is complexed with tubulin (Cai et al. 2011).

In this manuscript, we attempted to elucidate the interaction between CalS and microtubules as microtubules might control the insertion or activation of CalS into the plasma membrane, finally regulating pollen tube growth. We previously showed that the distribution of CalS in the distal region of tobacco pollen tubes depends on the integrity of microtubules. In addition, we have shown by Blue Native electrophoresis that CalS is associated with tubulin (Cai et al. 2011). However, the specific relationship between CalS and microtubules is still uncertain. In this work, we further investigated the connection between CalS and microtubules through a spin-down assay. In addition, we analyzed the molecular composition of the CalS complex both by native two-dimensional electrophoresis and by isolating the CalS complex using spin-trap and anti-CalS antibody. Subsequently, we studied the ultrastructural relationship between CalS and microtubules by immunogold labeling. Further information on the relationship between CalS and microtubules was obtained by far-western blotting while analysis of the association of CalS with soluble or insoluble fractions of membrane lipids showed that the enzyme is not uniformly distributed in cell membranes.

## Materials and methods

### Plants and reagents

Plants of *Nicotiana tabacum* (tobacco) were grown in the Botanical Garden of the University of Siena. Pollen was collected from closed anthers and induced to anthesis under controlled conditions. Pollen was dehydrated on silica gel and stored at − 20 °C. Before use, pollen was progressively acclimatized at room temperature (25 °C) and hydrated overnight in a moist chamber. Germination of pollen was achieved by resuspension in BK medium (at 1 mg/mL) supplemented with 12% sucrose (Brewbaker and Kwack 1963) for 3 h at the constant temperature of 25 °C. Tobacco seeds expressing GFP fused to Rab11b were kindly obtained from Prof. Alice Cheung (University of Massachusetts at Amherst, MA). Seed treatment and pollen collection have been already described (Cai et al. 2011). All chemicals and reagents were bought from Sigma Aldrich unless differently mentioned.

### Microtubule spin-down assay

**Fig. 1:**
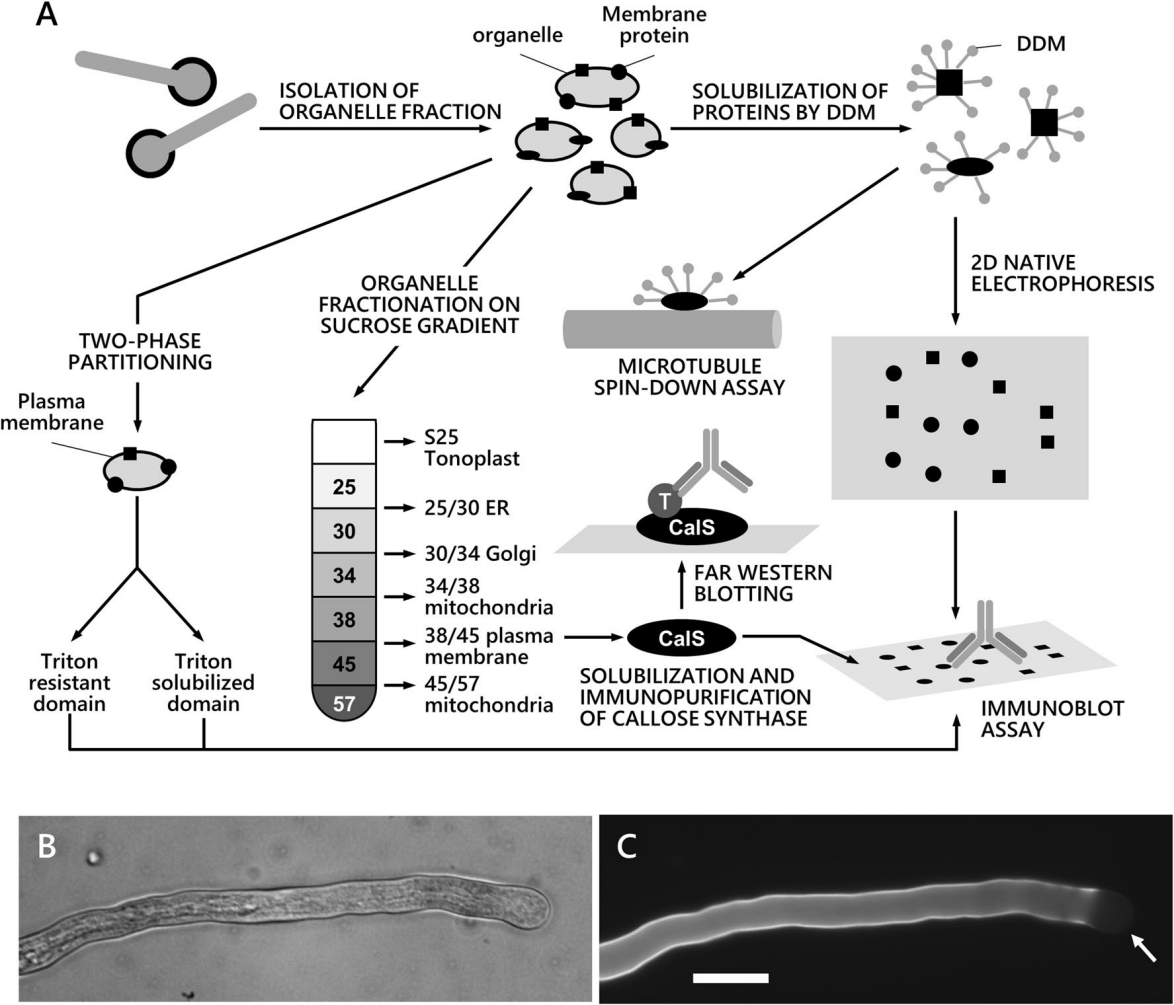
Outline of the biochemical analyses performed in this work and distribution of callose in tobacco pollen tubes. **A** Pollen tubes were subjected to membrane isolation; membranes were solubilized by DDM, and proteins were analyzed for their binding to microtubules in spin-down assays; in addition, proteins were separated by native 2-D electrophoresis, then transferred to nitrocellulose membranes and probed with antibodies. At the same time, the organelle pool of pollen tubes was fractionated on discontinuous sucrose gradient to obtain subcellular fractions enriched in specific classes of organelles. The plasma membrane fraction was solubilized by DDM and the callose synthase complex was purified by immunoaffinity. The purified CalS complex was subjected to far western blotting analysis with tubulin (T). Pollen tube membranes were subjected to two-phase partitioning to isolate the plasma membrane, which was then treated with Triton X-100 to isolate detergent-resistant domains. **B** DIC image of a tobacco pollen tube showing the tubular shape and multitude of organelles inside. **C** The same pollen tube shown in B labeled with the fluorescent dye Aniline Blue. Callose can be observed as a sheath surrounding the cell, except in the apical domain (arrow). Bar: 20 µm

The biochemical protocols used in this work are schematically summarized in Fig. 1A. For the microtubule spin-down assay, germinated pollen was washed in HEM buffer (50 mM Hepes pH 7.5, 2 mM EGTA, 2 mM MgCl_2_, 12% (*w*/*v*) sucrose) and resuspended in lysis buffer (50 mM Hepes pH 7.5, 2 mM EGTA, 2 mM MgCl_2_, 2 mM DTT, 10 µL/mL protease inhibitors, 10% *w*/*v* mannitol). Pollen was lysed on ice with a Potter–Elvehjem homogenizer and centrifuged at 10,000 g for 10 min at 4 °C and the low-speed supernatant was centrifuged at 100,000 g for 45 min at 4 °C on a 10% (*w*/*v*) sucrose cushion. The pellet (microsomal fraction, MF) was extracted with 2% (*w*/*v*) *n*‐dodecyl‐β‐D‐maltoside (DDM) in HEM buffer by incubating on ice for 50 min. Samples were centrifuged at 13,000 RPM for 60 min in a microfuge. The supernatant was stored on ice. Microtubules were prepared from unpolymerized tubulin (25 µL at 10 mg/mL) by adding 25 µL of TDB [PEM buffer (80 mM Pipes pH 6.8, 1 mM MgCl_2_, 1 mM EGTA) + 2 mM GTP + 20% (*v*/*v*) glycerol]. 6.25 µL of cushion buffer (PEM buffer + 20% sucrose) was also added and the sample was incubated at 35 °C for 20 min. At the end, the sample was supplemented with pre-warmed 400 µL of MDB-T (PEM buffer + 1 mM GTP + 37 µM taxol) to drop the tubulin concentration to 0.5 mg/mL. The sample was gently mixed and stored at room temperature. DDM-solubilized membrane proteins and microtubules were mixed under different conditions (± cytosol, ± 5 mM AMPPNP (adenyl-5′-imidodiphosphate)) and incubated for 30 min at room temperature. Samples were centrifuged at 100,000 g for 60 min at 25 °C, over a 20% sucrose cushion. Pellets and supernatants were analyzed by electrophoresis. The protocol is a slight modified version of the method described in http://www.bio-protocol.org/e980.

### Native bidimensional electrophoresis

Pollen was germinated in BK12S (12% sucrose in BK medium) for 2 h at 25 °C. The native bidimensional electrophoresis was performed according to a published method (Weiland et al. 2014) with minor modifications. After germination, the pollen was washed in 40 mM Bis–Tris pH 7.5 supplemented with 12% sucrose by centrifuging at 1000 RPM, 20 °C, 5 min. The final pollen pellet was resuspended in a lysis buffer (40 mM Bis–Tris pH 7.5, 2% (*v*/*v*) protease inhibitors) and lysed in a cold room using a Potter–Elvehjem homogenizer (40 strokes). The lysate was centrifuged at 10,000 g for 10 min at 4 °C. The supernatant was collected while the pellet was discarded. The supernatant was centrifuged again at 100,000 g for 45 min at 4 °C. The new pellet (containing pollen tube membranes) was resuspended with solubilization buffer (2% DDM, 40 mM Bis–Tris pH 7.5, 1% protease inhibitors). The sample was sonicated three times (1 min each) in cold water with intervals of 1 min. The sample was centrifuged at 15,000 g, 30 min, 4 °C; the resulting supernatant was supplemented with 2% IPG buffer and 1 mM DTT. IPG strips (pH range 3–10 NL, 11 cm) were placed over a proper sample volume using an IPG reswelling tray. Strips were covered with mineral oil and incubated overnight at room temperature. The IEF native electrophoretic run was performed with the following parameters: from 0 to 90 V in 2 h, from 90 to 180 V in 2 h, from 180 to 360 V in 2 h and 30 min, from 360 to 8000 V in 1 h and 30 min, 8000 V constant for 27,000 Vhr, 8000 to 180 V in 10 min, 180 V constant for 12 h. At the end, strips were used immediately or stored at − 80 °C. Before the second dimension, strips were incubated for 15 min in the SDS solubilization buffer (50 mM Tris–Cl, pH 8.8, 6 M urea, 30% glycerol, 2% SDS, trace of Bromophenol blue, 2 mM DTT) and then placed on top of gels.

### SDS-PAGE and immunoblotting

Proteins were separated by 1-D electrophoresis on pre-cast 10% Criterion XT gels (Bio-Rad) using a Criterion cell (Bio-Rad) powered by a Power Pac Bio-Rad 300 at 200 V for 45 min. Gels were stained with Bio-Safe Coomassie (Bio-Rad).

For immunoblot, proteins were transferred to nitrocellulose membranes using a Trans-Blot Turbo Transfer System (Bio-Rad). Membranes were blocked overnight at 4 °C in 5% ECL Blocking Agent (GE HealthCare) in TBS (20 mM Tris pH 7.5, 150 mM NaCl) containing 0.1% Tween-20. After washing in TBS, membranes were incubated with the primary antibody for 1 h. The antibody to CalS HDA is extensively described in the literature (Cai et al. 2011) and was used at the dilution of 1:300. To label actin, we used the mouse monoclonal anti-actin 10B3 (Sigma) diluted 1:3000. Sucrose synthase was detected using a rabbit polyclonal against maize sucrose synthase (Heinlein and Starlinger 1989) diluted 1:1000. Tubulin was labeled by the mouse monoclonal anti-tubulin B-5-1-2 (Sigma) diluted 1:5000. The anti-kinesin MMR44 (Marks et al. 1994) was used at the dilution of 1:200, while the anti-myosin at 1:1000 (Yokota and Shimmen 1994; Yokota et al. 1995). The anti-GFP antibody was obtained from Sigma Aldrich and used at a dilution of 1:1000. After washing with TBS, membranes were incubated for 1 h with peroxidase-conjugated secondary antibodies: an anti-mouse IgG (Bio-Rad, diluted 1:3000) and a goat anti-rabbit IgG (Βio-Rad) diluted 1:3000. After rinsing with TBS, immunological reactions were visualized with Immun-Star (Bio-Rad). Images of gels and blots were captured using a Fluor-S apparatus (Bio-Rad) and were analyzed with the Quantity One software (Bio-Rad).

### Organelle fractionation by sucrose gradients

The method is a slight modification of an already published protocol (Pertl et al. 2005). Pollen was germinated in BK12S medium for 3 h. After washing twice with HEM buffer, the pollen was resuspended in lysis buffer (HEM buffer plus 10 µL/mL protease inhibitors, 10% mannitol) and lysed on ice with a motor-driven Potter–Elvehjem homogenizer. The sample was centrifuged at 10,000 g for 10 min at 4 °C to remove large cellular debris and the low-speed supernatant was centrifuged again at 100,000 g for 45 min at 4 °C on a 10% (w/v) sucrose cushion. The pelleted microsomal fraction (MF) was resuspended in lysis buffer and separated into different organelle classes by a discontinuous sucrose gradient in HEM buffer plus 1 mM DTT and 1 mM PMSF. The gradient consisted of 1.7 mL of 25%, 30%, 34%, 38%, 45% and 57% sucrose. The different sucrose concentrations were layered into ultracentrifuge tubes using a peristaltic pump and a needle. The MF sample (max 1 mL) was loaded on top of the discontinuous sucrose gradient. Samples were centrifuged at 100,000 g for 2 h at 4 °C and the following organelle fractions were collected: S25, and the interfaces 25/30, 30/34, 34/38, 38/45, 45/57. All samples (except S25) were diluted to 10% (*w*/*w*) sucrose with HEM buffer and centrifuged at 100,000 g for 1 h 15 min at 4 °C. Pellets were resuspended in Laemmli Sample Buffer (LSB) for electrophoresis.

The obtained fractions were tested for the presence of four standard enzymatic markers of organelle identity, i.e., cytochrome C oxidase activity (CCO, for mitochondria), cytochrome C reductase (CCR, for endoplasmic reticulum), IDPase activity (for Golgi) and P-ATPase activity (for plasma membrane). The experimental protocols used are extensively described in the literature (Romagnoli et al. 2003).

### Isolation of callose synthase by spin trap

Membranes at the interface of step sucrose gradients were diluted with buffer and centrifuged to pellet membranes. Pellets were resuspended in the HEM buffer containing DDM to 1% final concentration (*w*/*v*). After incubation at room temperature for 1 h with agitation, samples were centrifuged (150,000 g, 60 min, 20 °C) over a 60% sucrose cushion and supernatants were directly applied to the Protein A Spin Trap column (GE HealthCare) according to the manufacturer’s instruction. The antibody solution (200 µL) was added allowing the CalS antibody to bind to Protein A. As a control, an anti-RubisCO antibody (Agrisera) was used in place of anti-CalS. The column was manually inverted and incubated with end-over-end mixing for 30 min. After centrifugation and washing for 1 min at 150 g to remove excessive unbound antibody, the protein sample was applied to the column and incubated with end-over-end mixing for 60 min. The column was centrifuged for 1 min at 150 g to wash out unbound proteins and then washed several times with the wash buffer. Proteins were eluted by adding 200 μL of elution buffer and mixing by inversion. Columns were centrifuged for 1 min at 1000 g and eluates were collected. After elution of proteins from the Protein A Spin Trap column, the pH was buffered by adding a volume of 1 M Tris (pH 8.0).

### Far Western blotting

This method was applied to the immuno-purified CalS complex. Proteins were denatured in LSB and separated by electrophoresis as described above; proteins were transferred to membranes and then underwent a process of denaturation/renaturation according to a protocol based on decreasing concentrations of guanidine-HCl (Yuliang et al. 2007). After overnight blocking, membranes were incubated with porcine tubulin (Cytoskeleton Inc.) at a concentration of 1 mg/mL in TBS supplemented with 0.1% Tween-20 (TBST) for 2 h at room temperature. After washing in TBS, membranes were incubated with primary anti-α-tubulin antibody B-5–1-2 (mouse) diluted 1:5000 in TBST or with antibody HDA (rabbit) against CalS diluted 1:300 in TBST. After a 1-h incubation at room temperature, membranes were washed in TBS and then incubated with secondary antibodies against mouse or rabbit immunoglobulins conjugated with HRP. Antibodies were used at a dilution of 1:5000 in TBST for 1 h at room temperature. After further washing in TBS, membranes were processed with the Clarity reagent (Bio-Rad) and the signal was detected with the Fluor-S device (Bio-Rad).

### Isolation of plasma membrane Triton-resistant micro-domains

Triton-resistant micro-domains were isolated from the pollen tube plasma membrane using protocols available in the literature (Bessueille et al. 2009; Srivastava et al. 2013). All steps were performed at 4 °C. After germination, pollen tubes were washed in washing buffer (50 mM Hepes pH 7.5, 2 mM EGTA, 2 mM EDTA, 12% sucrose), then lysed in lysis buffer using a Teflon Potter–Elvehjem homogenizer. The lysate was centrifuged at 1500 g, 4 °C for 5 min to remove cell debris. The resulting supernatant was centrifuged at 4 °C, 12,000 g for 20 min to remove mitochondria and other organelles. The resulting low-speed pellet (LSP) was processed for electrophoresis. The supernatant was centrifuged again at 4 °C, 100,000 g for 1 h, the resulting pellets being the total microsomal vesicles (MSV). The resulting high-speed supernatant (HSS) corresponded to the cytosolic fraction. The microsomal pellet was suspended in 2 mL of 5 mM KH_2_PO_4_ (pH 7.8). 1.8 g of MSV was added to 5.4 g of two-phase mixture, made by 5.8% (*w*/*w*) PEG-3350, 5.8% (*w*/*w*) dextran T-500, 4 mM KCl, 5 mM potassium phosphate (pH 7.6). Samples were mixed by reversing 30 times. The two phases were separated by centrifugation at 1500 g for 5 min. The upper phase was removed and supplemented with an equal weight of the lower phase solution taken from a large two-phase mixture. Samples were stirred 30 times then centrifuged at 1500 g for 5 min. The upper phase was diluted 3–5 times with dilution buffer (50 mM Hepes pH 7.5, 250 mM sucrose, 1 mM EDTA) and centrifuged at 100,000 g for 1 h at 4 °C. The pellet was resuspended in 900 µL of cold dilution buffer (sample PM). To prepare the micro-domains, Triton X-100 was added to the final concentration of 1% and incubated for 30 min on ice; sucrose was then added to the final concentration of 46%. The sample was placed on a three-step sucrose gradient made by 2 mL of 40%, 30% and 15% sucrose in Hepes buffer, pH 7.5. Sucrose gradients were centrifuged at 131,000 g for 20 h at 4 °C using a swinging rotor. The Triton-resistant micro-domains were recovered as a white band at the 15–30% interface while the Triton-solubilized proteins were collected at the tube bottom (in the 46% sucrose layer). Both samples were diluted 1:2 with Hepes buffer (50 mM, pH 7.5) and centrifuged at 100,000 g for 2 h at 4 °C. The two resulting pellets (MD for micro-domains, TP for Triton-solubilized proteins) were suspended in Hepes buffer (50 mM, pH 7.5).

### Immunogold electron microscopy

Immunogold electron microscopy was carried out as previously described (Cai et al. 2011). Primary HDA anti-CalS antibody was diluted 1:50. Secondary antibodies (anti-rabbit 15-nm gold conjugates) used in immunogold electron microscopy were from Agar Scientific and were diluted 1:50.

## Results

### Callose synthase does not bind to microtubules

Callose is a glucose polymer that forms a tubular sheath surrounding the pollen tube except at the apex. Most likely, callose strengthens the cell wall in those regions where growth should not occur. Consequently, the occasional presence of callose at the tube apex is a typical marker of growth cessation. The distribution of callose indicates that the polymer is synthesized at the level of the subapical region and remains present along the tube shank (Fig. 1B, C).

**Fig. 2:**
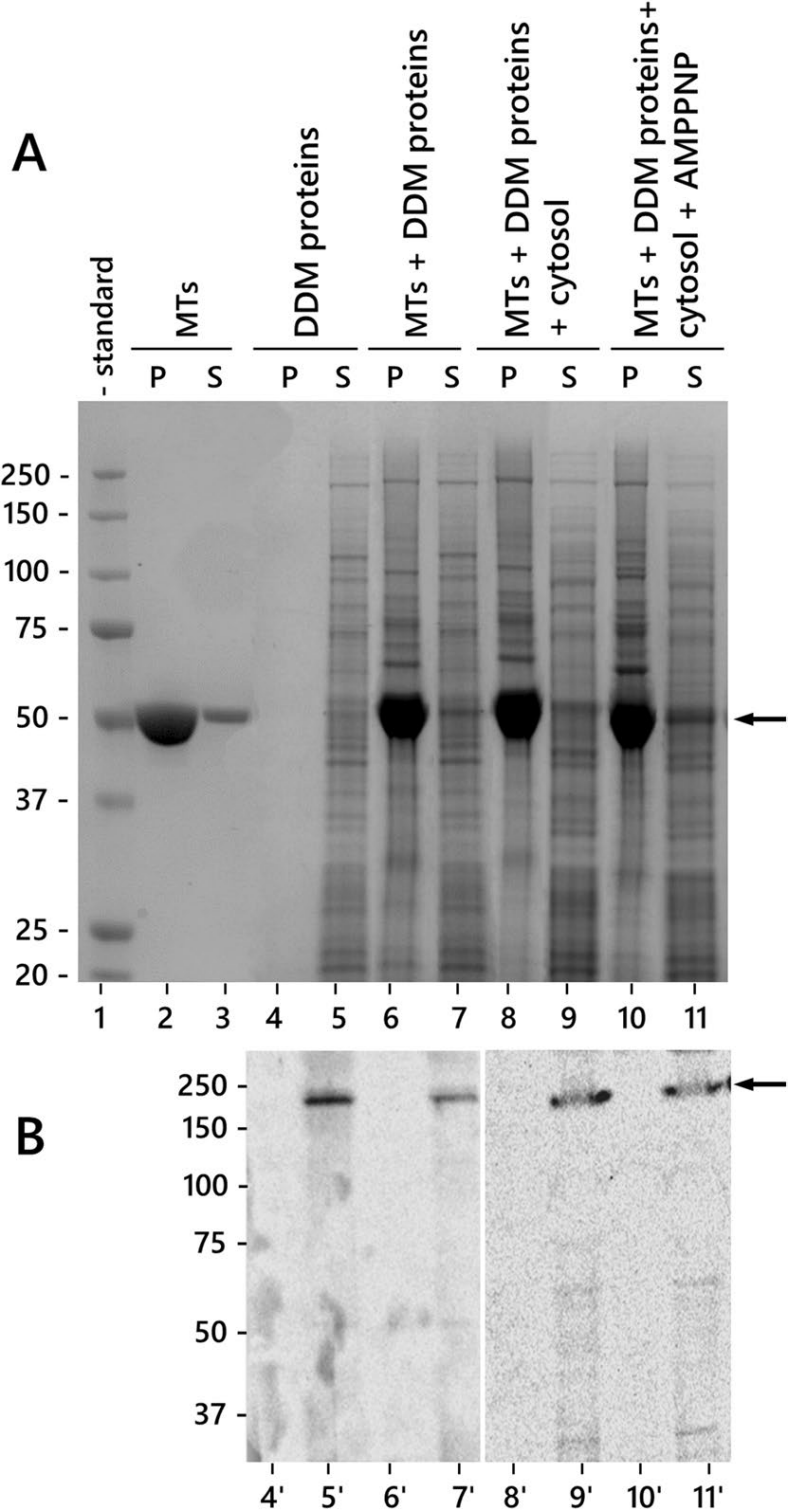
Microtubule and CalS spin-down assay. **A** SDS-PAGE analysis. Lane 1, molecular weight standards. Lanes 2–3, microtubules subjected to spin-down assay (P, pellet; S, supernatant). Lane 4–5, membrane proteins from tobacco pollen tubes do not sediment when subjected to spin-down assay. Lanes 6–7, membrane proteins mixed with microtubules and subjected to spin-down assay; some proteins are found in the microtubule pellet. Lane 8–9, mix of cytosolic proteins, microtubules, and membrane proteins subjected to spin-down. Lanes 10–11, membrane proteins mixed with microtubules and 5 mM AMPPNP subjected to spin-down assay. Arrow indicates the position of tubulin. **B** Immunoblot analysis with CalS antibody on some fractions shown in A. CalS is always found in the supernatant in the absence of microtubules (lane 5'), in the presence of microtubules (lane 7'), in the presence of microtubules and cytosolic proteins (lane 9), and after addition of 5 mM AMPPNP (lane 11'). Arrow indicates the immunoreactive CalS

To understand the relationships between CalS and microtubules, we initially analyzed whether CalS could bind to microtubules by spin-down assays. Microtubules alone were mainly found in the pellet (Fig. 2A, lane 2–3). Under the same experimental conditions, DDM-solubilized membrane proteins from tobacco pollen tubes did not precipitate and were therefore recovered in the supernatant (lane 4–5). Immunoblotting analysis showed that CalS was accordingly detected in the supernatant (Fig. 2B, lane 5'). When the membrane proteins were incubated with microtubules and then sedimented, some proteins were found in the pellet along with microtubules (lane 6–7); however, CalS was still detected in the supernatant (Fig. 2B, lane 7'). Cytosolic proteins were added to the membrane-microtubule mix to test whether soluble proteins could promote binding between CalS and microtubules (lane 8–9); again, CalS was found in the supernatant (lane 9). Finally, membrane proteins and microtubules were incubated with the addition of 5 mM AMPPNP (the latter promotes binding of motor proteins to microtubules) and sedimented (lane 10–11). Under these conditions, CalS was again found in the supernatant (Fig. 2B, lane 11'). Results suggested that CalS does not bind to microtubules, at least under the binding conditions used.

### Callose synthase and tubulin co-separated by 2D-native electrophoresis

**Fig. 3:**
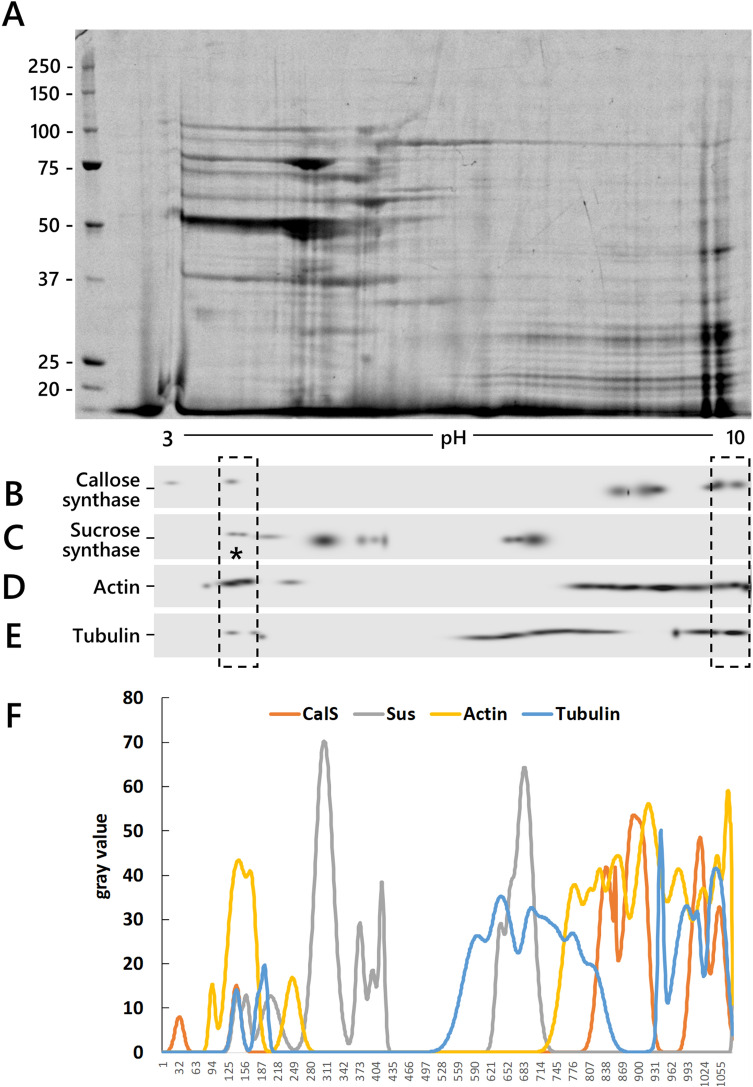
Native 2-D electrophoresis of membranes extracted from tobacco pollen tubes. **A** Typical electrophoretic pattern of membrane proteins separated using native 2-D gels. Molecular weight standards are shown on the left. The pH gradient is shown at the bottom. **B**–**E** Immunoblot on nitrocellulose membranes after protein transfer. The four proteins analyzed (CalS, sucrose synthase, actin, and tubulin) distribute differently than CalS; sucrose synthase is found in more defined regions, whereas actin and tubulin distribute throughout the pH gradient. Asterisk indicates the only portion of sucrose synthase that co-migrates with CalS. Blots are so-called “master blots” (i.e., the virtualization of real blots) that better define the number and location of individual immunoreactive spots. Images of the original blots are shown in Supplementary Fig. 1. **F** Measurement of CalS, sucrose synthase, actin, and tubulin distribution in representative immunoblots. Tubulin (blue line) is found in three regions: an acidic region at pH 4 and two more basic regions at pH 7–8 and pH 9–10. Actin (yellow line) accumulates consistently at pH 3–4 and is widely distributed from pH 6 to 10. Sucrose synthase (Sus, gray line) accumulates at pH 3–4 while a second peak is found at pH 5. A final peak of sucrose synthase is detected at pH 7. CalS (orange line) is detected as a small peak at pH 4 while two larger peaks are found in more basic regions at pH 9 and 10 (color figure online)

Native two-dimensional electrophoresis of membrane proteins from tobacco pollen tubes (Fig. 3) was used to test whether CalS and other proteins of interest co-separated as a molecular complex. This technique initially separated the native molecular complexes by IEF. These molecular complexes were further fractionated into the second dimension by denaturing electrophoresis. Membrane proteins were found throughout the 3–10 pH range (Fig. 3A). We specifically analyzed the distribution of four proteins (CalS, sucrose synthase, actin, and tubulin) by immunoblotting (Fig. 3B–E). Results were then further analyzed using QuantityOne software. Because the native IEF technique generates streaking in the signals that compromises spot identification, analysis through QuantityOne was used for spot identification between different blots. Hence, Figs. 3B–E show a "virtual" image generated by the software, highlighting identified spots. Four proteins showed a dissimilar distribution and CalS was found in distinct areas, with a peak around pH 4 and two additional peaks around pH 9 and 10 (Fig. 3B). This suggests that CalS may be associated with different protein complexes. The distribution of sucrose synthase was different from CalS, with the enzyme accumulating in regions corresponding to pH values 4, 5, and 7.5 (Fig. 3C). Only a subfraction of sucrose synthase co-separated with CalS (Fig. 3C, asterisk). The distribution of actin and tubulin was broader than that of CalS and sucrose synthase. The most acidic peak of actin (around pH 4) co-distributed with both CalS and sucrose synthase, whereas the rest of the actin covered a basic pH range around 8–10 (Fig. 3D). The migration pattern of tubulin could be divided into three regions, a more acidic region (around pH 4), a neutral region at pH 6.5–8.5, and a third basic region at pH 8.5–10. The tubulin fraction at acidic pH corresponded to the elution profile of CalS, sucrose synthase, and actin. The basic peak (around pH 9–10) of tubulin corresponded to the basic peak of CalS (Fig. 3E).

Protein co-fractionation was more evident after software analysis of immunoblotting profiles (Fig. 3F). Analysis with QuantityOne confirmed that tubulin (blue line) distributed in three regions, two of which (the acidic region at pH 4 and the basic region at pH 9–10) overlapped with the CalS profile (orange line) (Fig. 3F, arrows). Actin (yellow line) accumulated around the acidic peak of CalS (pH 4) but was widely distributed in the basic region, thus overlapping the peak of tubulin and CalS. Sucrose synthase (gray line) co-distributed with tubulin, CalS, and actin peaks in the acidic region at pH 4, but also showed an unrelated distribution at pH 5–6. CalS, sucrose synthase, actin, and tubulin aligning in the pH 4 region might indicate a single protein complex. Some degree of overlap between tubulin and CalS also occurred around pH 10.

### Tubulin and callose synthase co-fractionated in the plasma membrane fraction

**Fig. 4:**
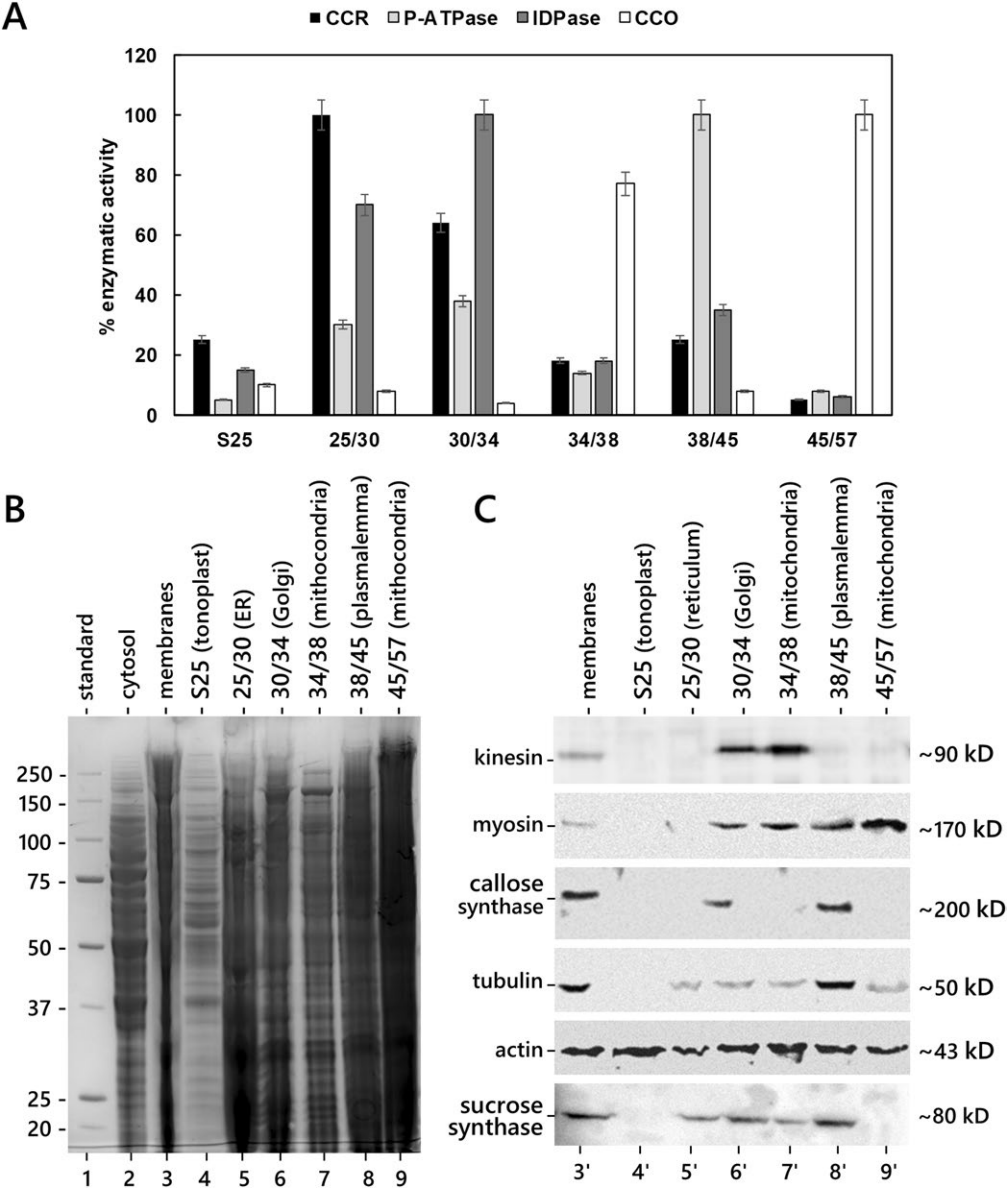
Biochemical and immunological characterization of organelle fractions from pollen tube. **A** Analysis of marker enzymes for specific organelle classes. Enzyme activities are expressed as percentage activity. CCR: cytochrome C reductase; CCO: cytochrome C oxidase. The x-axis shows the interfaces of the discontinuous sucrose gradient at which fractions were found. **B** Electrophoretic analysis of membrane fractions from discontinuous sucrose gradients (including cytosol). Molecular weight standards are on the left. The predominant content of each organelle fraction is shown at the top. **C** Immunoblots with different antibodies on the same organelle fractions shown in A (excluding cytosol). Proteins recognized by the antibodies are shown on the left. On the right, the molecular weight of the identified proteins

The relative distribution of CalS and tubulin was studied by enzymatic and immunological characterization of different organelle fractions (Fig. 4). The pool of total pollen tube membranes was fractionated by discontinuous sucrose gradient, resulting in six different subcellular fractions, each enriched in a specific organelle class (Fig. 4A). The S25 fraction was enriched in tonoplast. According to the marker assay, the interface 25/30 contained mainly endoplasmic reticulum (high cytochrome C reductase activity), while the interface 30/34 was enriched in IDPase activity, a marker of Golgi elements. The interface 34/38 contained a subfraction of mitochondria (as shown by cytochrome C oxidase activity), whereas a second subfraction of mitochondria was located at the interface 45/57. The plasma membrane fraction was found at interface 38/45, as shown by the high activity of P-ATPase.

We then analyzed the presence and distribution of specific proteins in organelle fractions (Fig. 4B, C). In addition to the previously analyzed proteins, we also investigated the distribution of two motor proteins (kinesin and myosin); the latter appeared to be distributed differently because kinesin was restricted to the light mitochondrial fraction and the Golgi fraction (Fig. 4C, lanes 6'-7'); the Golgi fraction showed two immunoreactive bands, perhaps corresponding to two distinct isoforms. Myosin was detected in association with all organelle fractions except the tonoplast (Fig. 4C, lanes 5'-9'); in this case, only a very faint band was evident. CalS was found in Golgi and plasma membrane fractions (Fig. 4C, lanes 6' and 8'), suggesting that it might be transported according to a standard secretory pathway. Tubulin was most consistently found in the plasma membrane fraction (lane 8') while the signal in the other fractions was weaker. In contrast, actin was largely present in all organelle fractions, supporting a role for actin in the organization of the endo-membrane system of pollen tubes. The enzyme sucrose synthase appeared to be associated with the endoplasmic reticulum/Golgi/plasma membrane (lanes 5', 6', and 8') suggesting a membrane-based delivery.

### Tubulin is part of the callose synthase complex

**Fig. 5:**
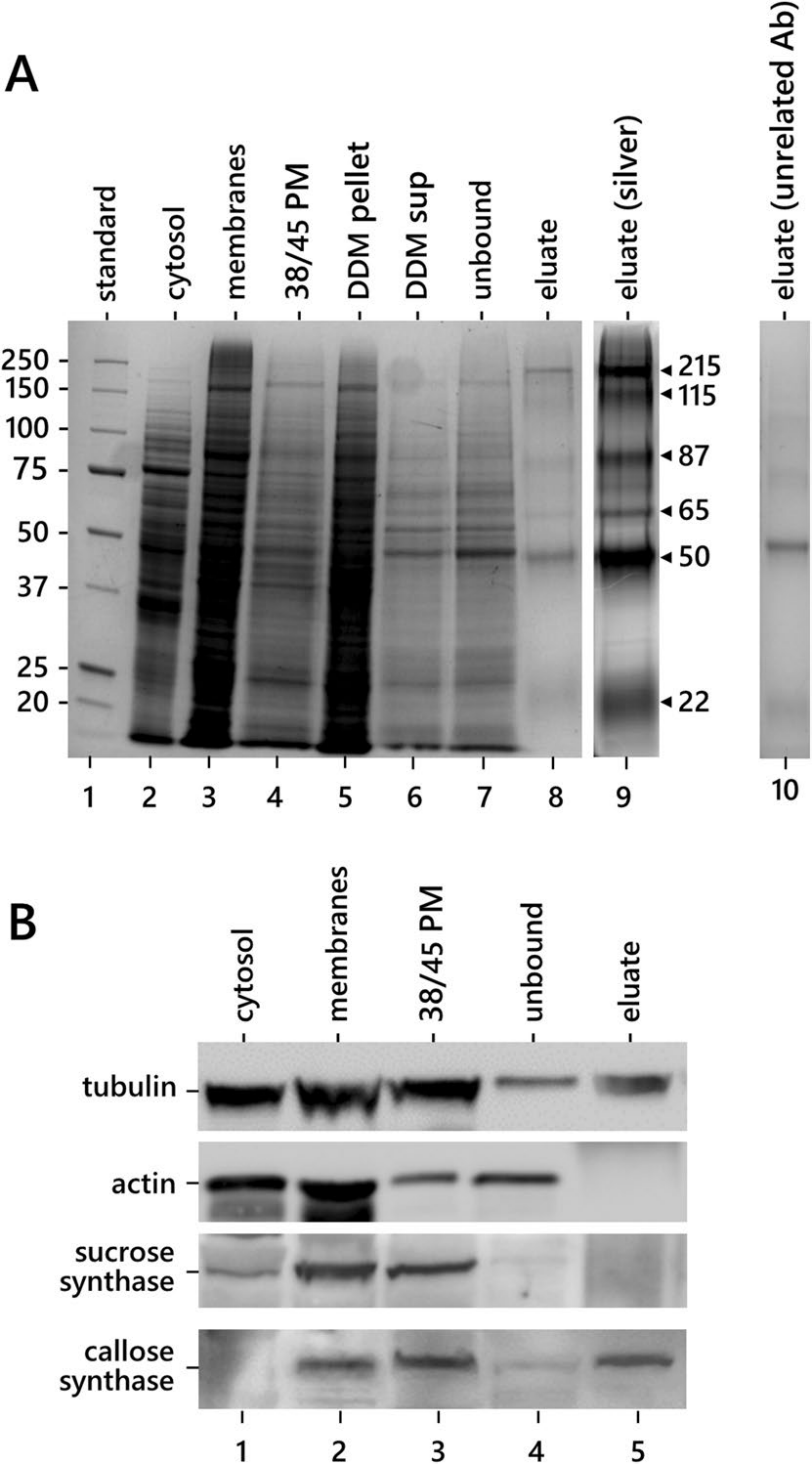
CalS immunosorbent assay. **A** Electrophoretic analysis of main fractions obtained during CalS immunopurification. Lane 1, molecular weight standards. Lane 2, cytosol. Lane 3, total membrane fraction. Lane 4, 38/45 fraction (enriched in plasma membrane protein). Lane 5, membrane pellets after protein extraction by DDM. Lane 6, proteins eluted by DDM treatment. Lane 7, proteins that do not bind to the CalS antibody. Lane 8, proteins that bind to the CalS antibody and then eluted. Lane 9, same sample as lane 8 after silver staining. Lane 10, eluate obtained after the use of an unrelated antibody (not directed against CalS). **B** Immunoblots on some representative fractions described in A. Proteins recognized by the antibodies are indicated on the left. The final sample (eluate) contains relevant amounts of tubulin and CalS

To further investigate the hypothetical interaction between CalS and tubulin, we investigated the presence of tubulin in the CalS complex using an immunosorbent assay (Fig. 5). The CalS complex was isolated by immuno-purification using a Protein A Spin Trap column (Fig. 5A). The total membrane fraction (lane 3) was fractionated through the discontinuous sucrose gradient by obtaining the 38/45 fraction (lane 4) enriched in plasma membrane proteins. The 38/45 fraction was then extracted with DDM detergent obtaining a pellet of unsolubilized proteins (lane 5) and a supernatant of DDM-solubilized membrane proteins (lane 6). The latter sample was loaded onto a Protein A Spin Trap column, on which CalS antibodies had been previously absorbed. Proteins not bound to the antibody were removed by washing (lane 7), whereas proteins bound to the CalS antibody were eluted (lane 8). Proteins bound to the CalS antibody were also stained with silver to improve visualization (lane 9). As a control, an unrelated antibody against Rubisco heavy chain was used (lane 10). The eluted sample presented several polypeptides, the most prominent at 215, 115, 87, 65, 50, and 22 kD. The band at 215 kD corresponded to CalS (Fig. 5B).

Fractions described above were analyzed by immunoblotting against tubulin, actin, sucrose synthase, and CalS (Fig. 5B). As expected, tubulin was found in the cytosolic fraction (lane 1) but also in the total membrane fraction (lane 2), as well as in the plasma membrane fraction (lane 3). A substantial amount of tubulin was also detected in the final eluate (i.e., the protein complex bound to the CalS antibody, lane 5). In contrast, actin was not found in the final eluate even though it was present in the total membrane pool (lane 2). Sucrose synthase was found in the plasma membrane faction (lane 3), but not in the bound or eluted protein samples (lanes 4–5). CalS was detected in the total membrane fraction (lane 2) and in the plasma membrane fraction (lane 3); therefore, it bound to the antibody column and was eluted consistently in the final sample. The results showed that tubulin and CalS strongly associate and that therefore tubulin is part of the CalS complex.

**Fig. 6:**
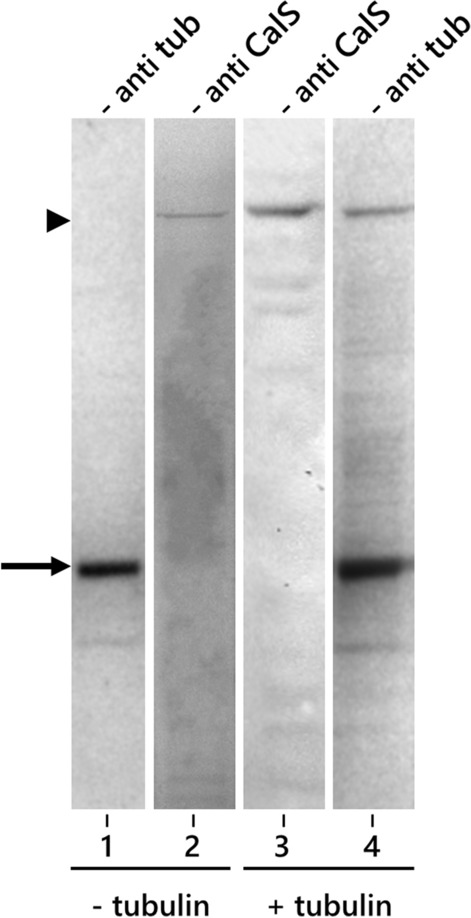
Far western blotting using tubulin as a bait protein to identify tubulin-binding proteins in the CalS complex. Lane 1, labeling with anti-tubulin without pre-incubation with tubulin; lane 2, labeling with anti-CalS without pre-incubation with tubulin; lane 3, labeling with anti-CalS after pre-incubation with tubulin; lane 4, labeling with anti-tubulin after pre-incubation with tubulin. The arrow indicates the position of tubulin, the arrowhead indicates the position of CalS

As a further assay of binding between tubulin and CalS, we investigated their interaction by far-western blotting (Fig. 6) using tubulin as a bait protein for the previously isolated CalS complex. In the absence of tubulin as a bait protein, the anti-tubulin B-5-1-2 antibody selectively labeled tubulin in the purified sample (lane 1, arrow) whereas the anti-CalS antibody specifically marked the high molecular weight band of the enzyme (lane 2, arrowhead). In membranes incubated with tubulin as bait before antibody labeling, the anti-CalS antibody detected only the high-molecular-weight band of the enzyme (lane 3), whereas the anti-tubulin antibody detected both the 50-kD tubulin band and the 200-kD CalS band (lane 4). This finding indicated that tubulin is associated with CalS.

### Callose synthase is associated with Triton-resistant micro-domains of the plasma membrane

**Fig. 7:**
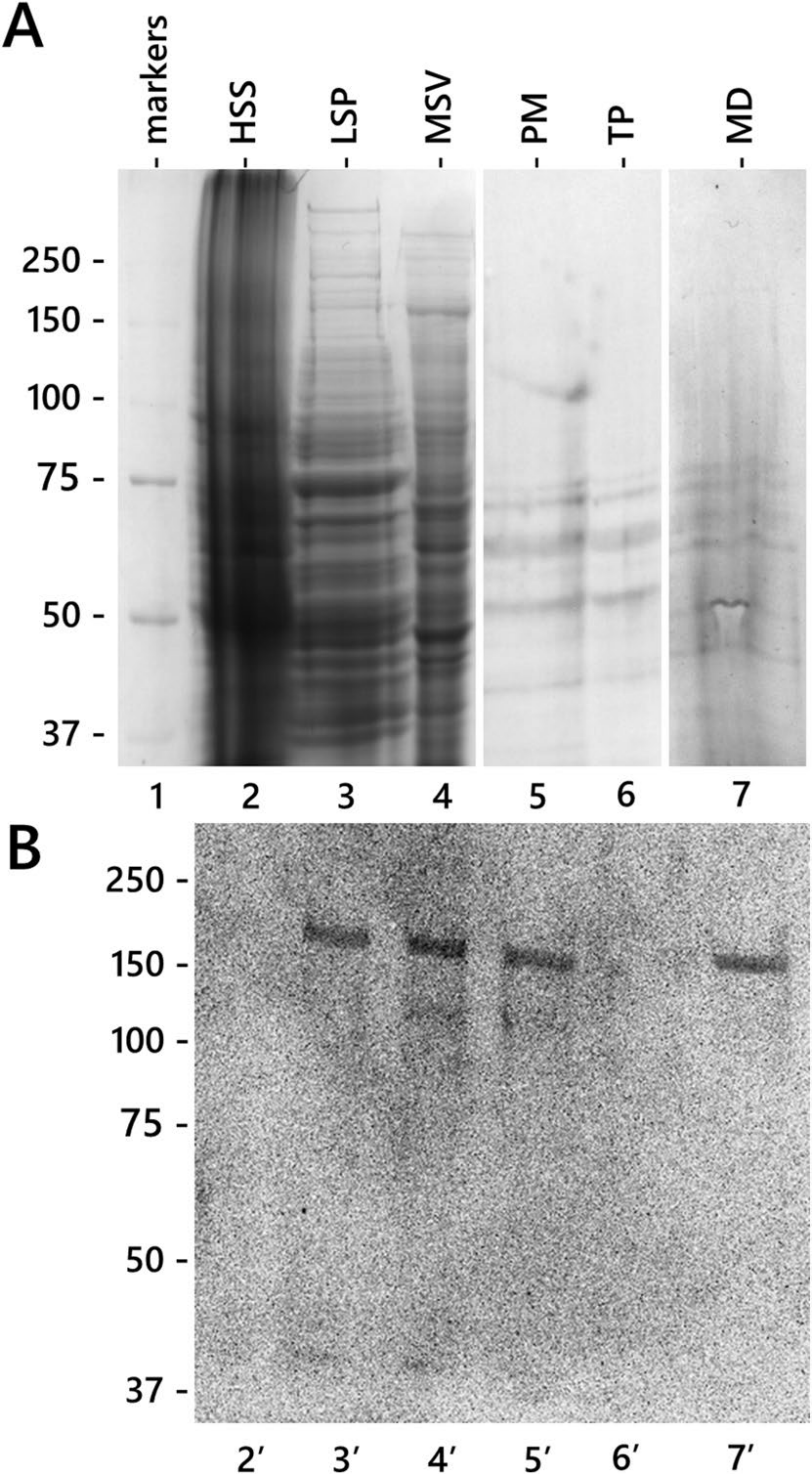
Immunoblotting with CalS antibody on fractions obtained after plasma membrane isolation by two-phase partitioning and solubilization with Triton X-100. **A** Gel electrophoresis of main fractions. Lane 1, standards of molecular weight. Lane 2, high-speed supernatant (HSS-cytosol). Lane 3, low-speed pellets (LSP). Lane 4, microsomal vesicles (MSV). Lane 5, plasma membrane (PM). Lane 6, Triton-solubilized proteins (TP). Lane 7, proteins not solubilized by Triton (MD, microdomains). **B** Immunoblot with anti-CalS antibody. Lane 2’, high-speed supernatant (HSS-cytosol). Lane 3’, low-speed pellet (LSP), i.e., total organelle fraction. Lane 4’, microsomal vesicles (MSV), the fraction of membranes without larger organelles. Lane 5’, plasma membrane (PM). Lane 6’, plasma membrane proteins solubilized by Triton (TP). Lane 7’, proteins not solubilized by Triton (MD, microdomains). An equal amount of protein was loaded into each lane

Previous results indicate that tubulin and CalS may interact in a single molecular complex probably located in the plasma membrane. We, therefore, decided to analyze whether CalS was associated with specific areas of the plasma membrane. Specifically, we investigated the presence of CalS in lipid regions solubilized or not by Triton (Fig. 7A, B). We first isolated the plasma membrane of pollen tubes by two-phase partitioning (PM, lane 5), then this fraction was processed with Triton detergent, and the Triton-solubilized (TP, lane 6) and Triton-resistant subfractions (MD, lane 7) were separated (Fig. 7A). Analysis by immunoblotting with the anti-CalS antibody (Fig. 7B) showed the absence of CalS in the cytosolic fraction (HSS, lane 2’); however, CalS was associated with the microsomal fraction (MSV, lane 4’) and the plasma membrane fraction (PM, lane 5’). More importantly, the CalS signal was also found in the Triton-resistant lipid fraction (MD, lane 7’) but not in the Triton-solubilized fraction (TP, lane 6’).

### Callose synthase associates with RAB11b-labelles vesicles

**Fig. 8:**
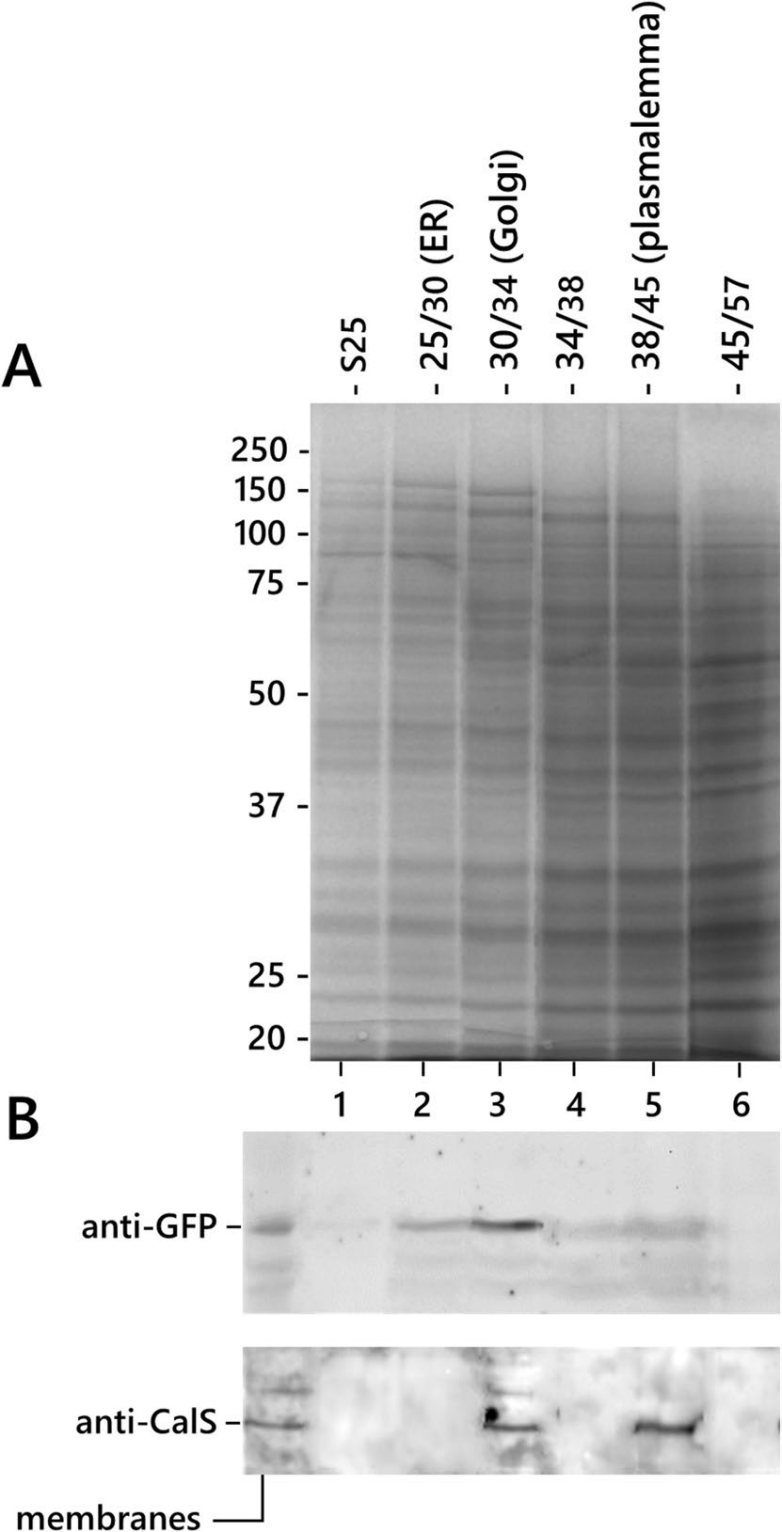
Relative distribution of GFP-tagged Rab11b and CalS. **A** Electrophoretic analysis of membrane fractions obtained from pollen tubes after centrifugation along sucrose step gradients. Samples are indicated at the top according to the corresponding interface. Molecular weight standards are on the left. **B** Immunoblots on the same fractions with antibodies against GFP and CalS. Panel B also contains the total membrane fraction (first lane on the left). GFP (and thus Rab11b-containing vesicles) were found in fraction 25/30 (containing the endoplasmic reticulum) and fraction 30/34 (containing the Golgi membranes). In contrast, CalS is found in the Golgi (30/34) and plasma membrane (38/45) fractions

After analyzing the distribution of CalS in the membrane compartments, we focused on Golgi-derived secretory vesicles, analyzing the relative distribution of CalS and Rab11b (Fig. 8). Rab11b is associated with Golgi-derived membranes and localizes to the tube apex. The relative distribution of Rab11b and CalS was studied after extraction of proteins from transgenic pollen tubes constitutively expressing GFP-tagged Rab11b and immunoblotting with anti-CalS and anti-GFP antibodies. Membranes were extracted and fractionated as described above along discontinuous sucrose gradients (Fig. 8A). Immunoblotting with anti-GFP and anti-CalS antibodies showed that GFP (and thus Rab11b-containing vesicles) localized in the 25/30 fraction (i.e., the endoplasmic reticulum) but more consistently in the 30/34 fraction (containing Golgi membranes) (Fig. 8B). CalS was found in the Golgi fraction (30/34) and in the plasma membrane fraction (38/45). Therefore, Rab11b and CalS co-localize in the Golgi fraction suggesting that CalS may be delivered by a conventional secretory pathway to the tube apex and inserted into the plasma membrane.

### Callose synthase is present in secretory vesicles and co-distributes with cortical microtubules

**Fig. 9:**
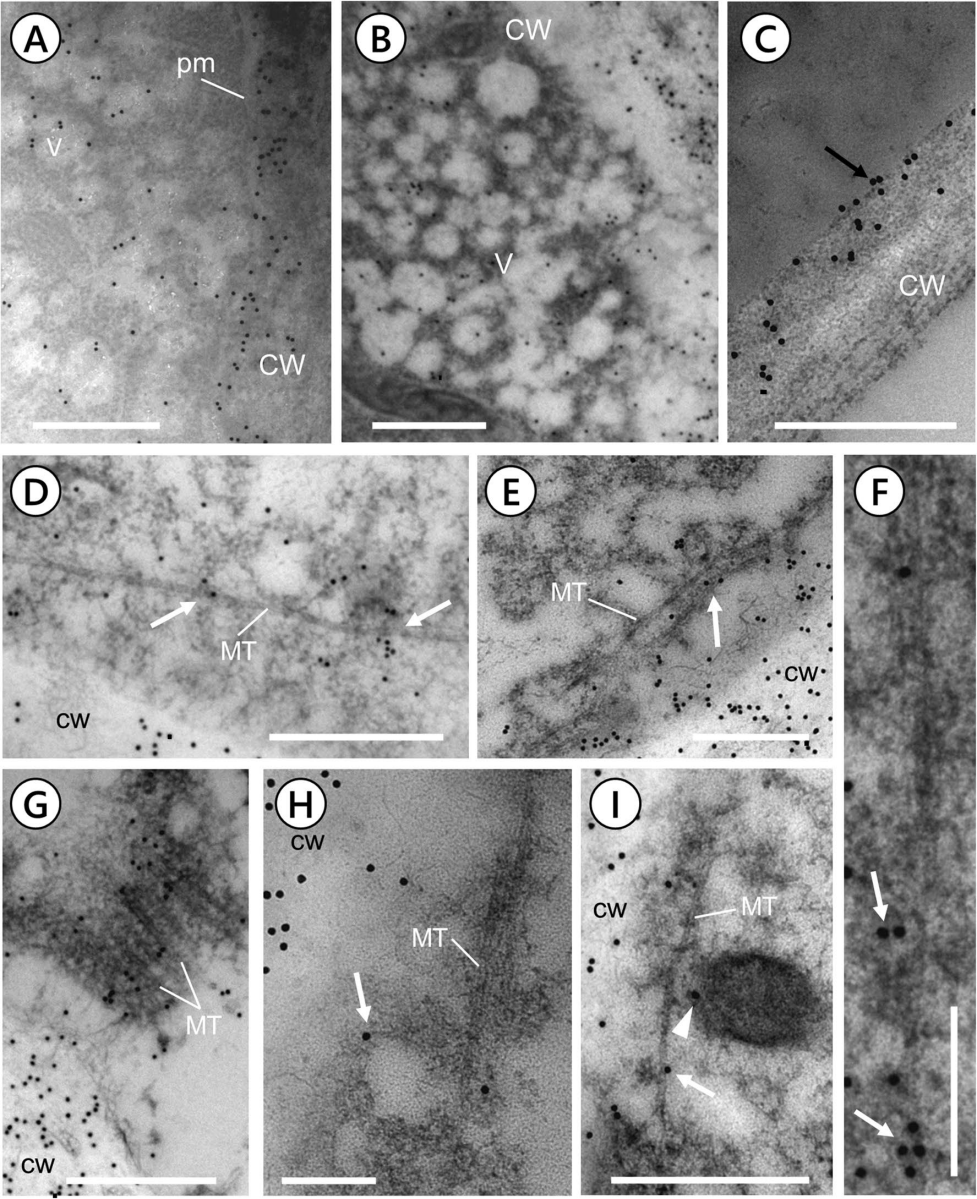
Localization of CalS in the pollen tube by immunogold electron microscopy. Images A-B were taken in the tip domain, and image C was taken in distal regions of pollen tubes. **A** The tip domain of pollen tubes contains several small vesicles (v) labeled by gold particles. Vesicles are secretory intermediates that release CalS into the apical plasma membrane (pm). Bar: 400 nm. **B** Another view of the apical domain showing secretory vesicles labeled by gold particles. Bar: 400 nm. **C** Gold labeling in the distal region of pollen tubes showing that CalS accumulates at the plasma membrane and cell wall. Bar: 400 nm. **D** A section in the cortical region of pollen tubes at 50–100 µm from the tip. The section shows a microtubule (MT) near the plasma membrane and cell wall (containing several gold particles). Aggregates of gold particles (arrows) are also near the microtubule. CW, cell wall. Bar: 500 nm. **E** A similar section from a different pollen tube showing microtubules below the plasma membrane and labeled by gold particles (arrow). Bar: 400 nm. **F** Some microtubules under the plasma membrane and labeled by different gold particles (arrows); bar: 200 nm. **G** Another section under the plasma membrane of a pollen tube. Several parallel microtubules are decorated with gold particles. In the lower left corner, a portion of the cell wall containing several gold particles. Bar: 600 nm. **H** A cortical section showing a microtubule (MT) in relation to a vesicle labeled by gold particles (arrow). Bar: 200 nm. **I** Image of a microtubule (MT) near the plasma membrane. A portion of the cell wall is shown on the left. The microtubule is associated with gold particles but also with vesicles labeled by gold particles (arrows). Bar: 400 nm

The relationship between CalS and secretory vesicles was further investigated by immunogold labeling. In the apical domain of pollen tubes, vesicles were found to be decorated with gold particles indicating the presence of CalS (Fig. 9). Notably, in the apical cortical domain (Fig. 9A) several gold particles were observed adjacent to secretory vesicles (V) and in association with the plasma membrane (pm) of pollen tubes (right side; CW: cell wall). The vesicles are most likely involved in secretory processes that release CalS into the apical plasma membrane. Anti-CalS-labeled secretory vesicles were also observed in Fig. 9B (a detail of the apical domain). In contrast, in the distal regions of pollen tubes (Fig. 9C), CalS was detected only in the plasma membrane (arrow) and cell wall (CW).

Immunogold electron microscopy was also used to investigate the relationships between CalS and microtubules in tobacco pollen tubes (Fig. 9 D–I). In the cortical domains of pollen tubes, 50–100 µm from the tip (Fig. 9D), microtubules (MT) were observed near the plasma membrane and consequently the cell wall. Gold particles (i.e., CalS) were often found around microtubules or aligned along with them (arrows). CalS was also visualized in association with the cell wall (CW). The association of CalS with microtubules was a commonly observed feature; sometimes CalS (arrow in Fig. 9E) was interspersed with microtubules (MT). Aggregates of CalS were often observed near the microtubules below the plasma membrane. The density of gold particles increased in correspondence with microtubule bundles (as in Fig. 9F). Microtubules (MT) located just below the plasma membrane of the pollen tube and arranged in parallel were strongly decorated by CalS (Fig. 9G). The enzyme was also found to be present in the cell wall (CW). Gold particles were detected both in direct association with microtubules and in association with membranous structures (possibly involved in secretion) attached to microtubules (arrow in Fig. 9H). On closer inspection, Fig. 9I shows a cortical section of a pollen tube with a single microtubule (MT) near the plasma membrane and in contact with a gold-labeled vesicle (arrow). The microtubule was also tightly bound to other molecules of CalS (arrow).

## Discussion

In this manuscript, we report evidence supporting the association between CalS and tubulin in tobacco pollen tubes. Literature does not report a direct association of CalS with microtubules and the data obtained in our investigation in fact show that CalS does not bind directly to microtubules but forms a complex with monomeric tubulin. Although the implication of this binding is not readily clear, the evidence of co-localization between CalS and microtubules strengthens the hypothesis that the CalS/tubulin interaction may facilitate microtubule-dependent CalS distribution. The interaction does not require active kinesin-mediated transport, because this motor protein does not co-distribute with CalS. In contrast, the presence of CalS in Rab11b-containing vesicles suggests that CalS transport to the apical plasma membrane is dependent on the actin/myosin-based motor system. In the plasma membrane, CalS is not uniformly distributed but is associated with specific lipid domains of the plasma membrane.

Comparing the behavior of CalS with that of cellulose synthase, cellulose synthesis indeed requires glycosyltransferase to interact at least temporarily with microtubules (Paredez et al. 2006; Gutierrez et al. 2009; Lei et al. 2014). The evidence that CalS does not bind to microtubules could be explained by the lack of accessory proteins essential for binding, although the same results were obtained after the addition of cytosolic proteins or AMPPNP (the latter required for binding ATP-hydrolyzing proteins such as motor proteins). If the binding between CalS and microtubules is not direct, then CalS might be associated with other membrane proteins capable of interacting with microtubules. Sucrose synthase is likely one of the proteins that interacts with CalS (Salnikov et al. 2003), but there is no evidence that sucrose synthase can bind to microtubules (Cai et al. 2011).

To obtain more information about the ability of CalS to interact with other (cytoskeletal) proteins, we adopted a different strategy based on native IEF (O’Farrel 1975). The data indicate that CalS exists in three major protein charge variants, the more acidic one co-distributing with other proteins while the more basic one co-distributing with tubulin and actin. This information strengthens the hypothesis that CalS may interact with sucrose synthase (Amor et al. 1995; Salnikov et al. 2003), but the data also suggest that tubulin may be part of the CalS complex (Aidemark et al. 2009; Cai et al. 2011). Suggestively, the interaction at acidic pH could be evidence that protein binding occurs in a more acidic environment such as the cell wall.

To verify the association or co-distribution of CalS with other proteins (possibly cytoskeletal), we tested organelle fractions of the pollen tube. Fractionation of the pollen tube membranes allowed the identification of organelles enriched in CalS (Pertl et al. 2005) and the identity of the organelle subfractions was confirmed by enzymatic assays. CalS has been identified in association with Golgi and plasma membrane (Turner et al. 1998; Brownfield et al. 2008); in addition to CalS, the Golgi membrane fraction contained kinesin, myosin, actin, and sucrose synthase while the plasma membrane fraction contained myosin, tubulin, actin, and sucrose synthase. The association of sucrose synthase with Golgi membranes and plasma membrane is logical because it reiterates the need to produce UDP-glucose for the CalS complex (Hong et al. 2001).

The CalS complex contains proteins with molecular weights between 20 and 200 kD; among these we have identified tubulin but neither actin nor sucrose synthase. This suggests that sucrose synthase is not permanently bound to CalS, while on the contrary tubulin is more stably associated with the CalS complex. Comparing the polypeptide CalS composition of *N. alata* pollen with that of *N. tabacum*, several polypeptides show similar molecular weight. In *N. alata*, however, tubulin has not been described as part of the CalS complex (Brownfield et al. 2007).

The binding between tubulin and CalS is also confirmed by far-western blotting. Even with technical limitations (Yuliang et al. 2007; Miernyk and Thelen 2008), the far western blotting method is an accepted approach to establish protein interactions. Collectively, our results indicate that tubulin is part of the CalS complex, and it can interact with CalS. The association between tubulin and CalS raises the question about its biological implication. No data on similar models are available in the literature. Tubulin can interact with a multitude of different proteins; for example, several cell wall-related proteins were identified in extracts of *Arabidopsis* (Chuong et al. 2004). Tubulin-binding proteins fall into several classes, such as signaling, metabolism, translation, standard MAPs, and RNA-binding proteins; the variety of protein classes is evidence that tubulin can interact with different cellular proteins. However, tubulin-interacting proteins are also capable of binding microtubules. There is little evidence that the tubulin monomer is part of larger protein complexes. Tubulin can interact with stathmin to form a protein complex that destabilizes microtubules by inhibiting the availability of tubulin (Niethammer et al. 2004). Some additional information is available for animal systems, where intracellular chloride channel (CLIC) proteins are associated with protein partners including dynamin, α-tubulin, β-actin, creatine kinase, and 14-3-3 (Suginta et al. 2001). HSP90 can bind to tubulin forming a complex that likely controls the microtubule polymerization mechanism (Garnier et al. 1998). In the above cases, there is consensus that the tubulin-containing complex can regulate the intracellular position of the protein partner or be used to regulate microtubule dynamics. There are very few reports of interactions between CalS and tubulin (Aidemark et al. 2009; Cai et al. 2011) and are not conclusive as to why the association occurs. As an indirect comparison, similar studies on cellulose synthase can be considered. Immunoprecipitation of cotton fiber extracts with antibodies against GhCESA8 revealed several proteins associated with cellulose synthase, including CalS, sucrose synthase, and β-tubulin (Li et al. 2016). The presence of tubulin in association with cellulose synthase is thought to be important in regulating the orientation and deposition of β-1,4-glucans in the cell wall. Even without direct evidence, we support the hypothesis that tubulin in the CalS complex mediates binding between CalS and microtubules and thus promotes proper deposition of glucans.

Immunolocalization data support the association between CalS and microtubules in the cortical region of pollen tubes, both near the apex and near the callose plugs (Cai et al. 2011). In the latter case, CalS appears to be directly associated with microtubules but also with membranous structures linked to microtubules and potentially involved in CalS delivery. These observations suggest that microtubules participate in (or otherwise control) CalS distribution in the cortical region. Previously, it was found that the microtubule inhibitor oryzalin causes an uneven distribution of CalS in the distal regions of pollen tubes (Cai et al. 2011). Consequently, all of this suggests that microtubules control the positioning of CalS in specific regions of the pollen tube and that this control mechanism requires the presence of monomeric tubulin in the CalS complex. At the same time, actin filaments (essential for CalS transport in the plasma membrane) participate in callose synthesis by regulating the association of sucrose synthase with the plasma membrane. This is supported by evidence that actin inhibitors interfere with the localization of CalS in pollen tubes and that actin binds sucrose synthase (Winter et al. 1998; Cai et al. 2011). The subcortical scaffold of microtubules and actin filaments would then provide support through which CalS and sucrose synthase interact thereby promoting callose synthesis.

Tubulin present in the CalS complex could promote the interaction between microtubules and vesicle-associated CalS. This association could also be mediated by motor proteins; for example, the 90-kD kinesin identified by MMR44 (Cai et al. 2000) is associated with cortical microtubules, has microtubule-stimulated ATPase activity and moves microtubules. Fractionation of pollen tube membranes has demonstrated the presence of kinesin in association with Golgi membranes and mitochondria, which is also supported by earlier evidence (Wei et al. 2005, 2009; Lu et al. 2005). Nevertheless, like in the case of cellulose synthase, kinesins have not been found directly associated with the CalS complex; it is likely that these motor proteins do not play a direct role in the movement of glucan synthases along the plasma membrane but help regulate the process (Zhu et al. 2015; Kong et al. 2015) probably through stabilization of cortical microtubules (Ganguly et al. 2020).

Immunogold labeling data suggests that CalS is transported to the apex in association with secretory vesicles. Fusion of vesicles results in the insertion of the enzyme into the plasma membrane. These data are supported by evidence that CalS is localized in Golgi membranes and then in the plasma membrane (Brownfield et al. 2008; Cai et al. 2011). The codistribution between CalS and Rab11b (a marker of vesicular secretion) (de Graaf et al. 2005) and the association of Rab11b with the endoplasmic reticulum and Golgi membranes further confirms that CalS is associated with secretory vesicles and transported toward the apical plasma membrane. Similar results are described for vesicle-associated CalS in trichomes (Kulich et al. 2018).

**Fig. 10:**
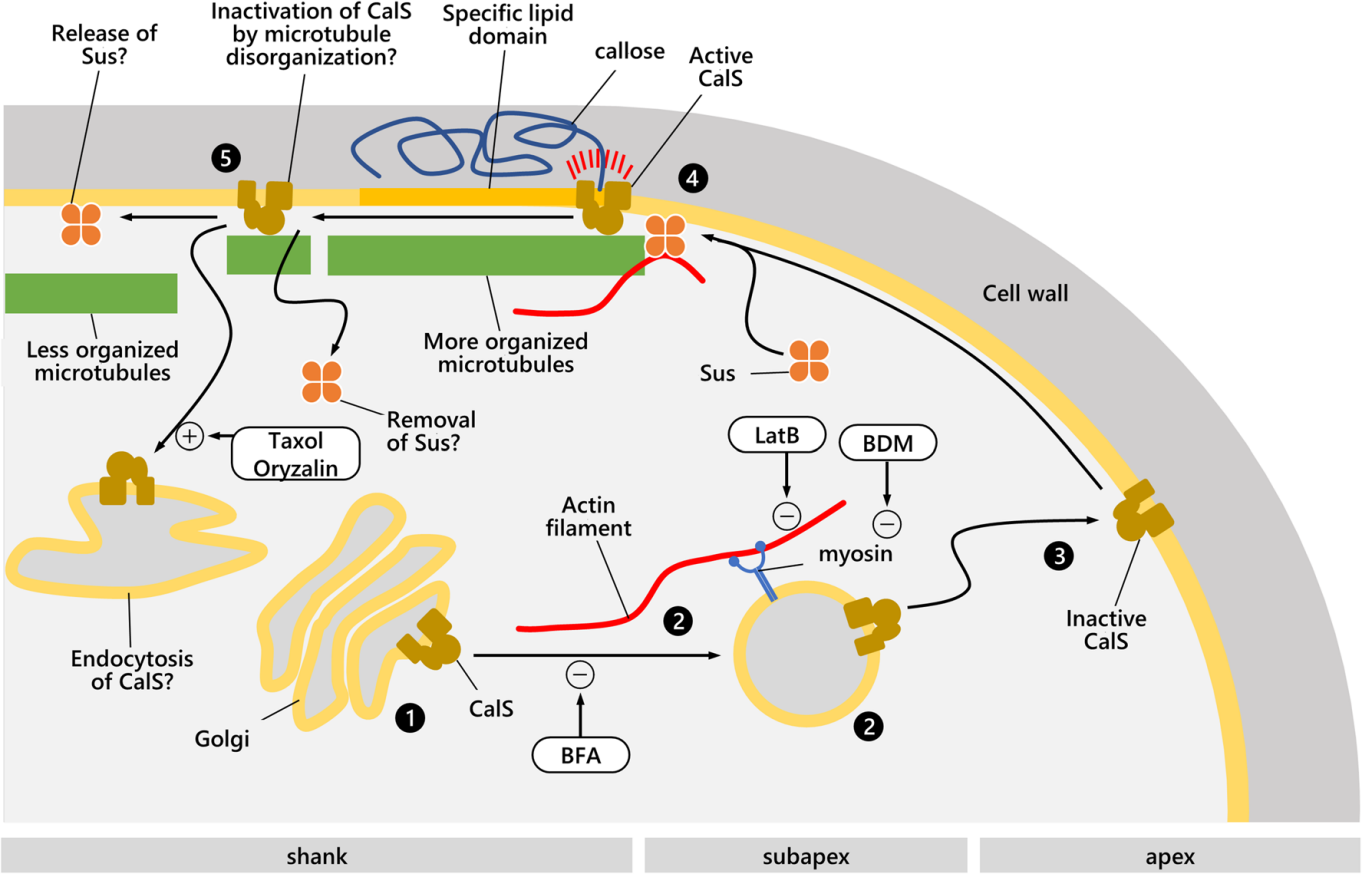
Outline of CalS trafficking and activation in relation to the cytoskeleton and membrane system. (1) CalS is synthesized in the endoplasmic reticulum (not shown), is moved to the Golgi, then (2) is transported by the apical secretion mechanism (powered by the actin/myosin motor system), and (3) inserted into the apical plasma membrane as inactive. When CalS, due to pollen tube elongation, is displaced into the subapex (4), it interacts with sucrose synthase (Sus) and is activated. A specific lipid composition of the membrane may promote activation. Tubulin binding may facilitate the association between CalS and microtubules and thereby regulate callose synthesis. In turn, the cortical positioning of Sus is dependent on actin filaments. In more distal regions, CalS is rendered inactive (5) perhaps by either different microtubule organization or by Sus release/removal or CalS internalization. The drawing shows the point of action of inhibitors that can promote (+) or inhibit (−) distinct steps in CalS transport. *BDM* 2,3-Butanedione monoxime; *LatB* Latrunculin B; *BFA* Brefeldin A

Overall, the data suggest a transport pathway for CalS in the pollen tube. First, CalS is produced in the endoplasmic reticulum, processed in the Golgi, and then exported via secretory vesicles. This latter step occurs along actin filaments (Cai et al. 2015; Madison et al. 2015). Secretory vesicles deliver inactive CalS to the apical plasma membrane (Brownfield et al. 2008). In the subapical region, CalS is activated, possibly by calcium ions (Aidemark et al. 2009) and Rop activity (Yamaguchi et al. 2006). Optimal levels of UDP-glucose could be provided by the transient association of CalS with sucrose synthase (Salnikov et al. 2003). Monomeric tubulin could be part of the mechanism by which CalS associates with cortical microtubules; this would promote microtubule-regulated callose synthesis or deposition. In more distal parts, CalS would be again inactivated, possibly because of the altered organization of cortical microtubules. Indeed, microtubule inhibitors such as taxol and oryzalin quantitatively affect callose synthesis (Aidemark et al. 2009) and promote CalS internalization (Cai et al. 2011) (Fig. 10).

Previous immunolocalization data have revealed that CalS is not uniformly distributed in the plasma membrane but rather associated with specific regions (Cai et al. 2011); this might suggest that the functioning of CalS depends on a specific lipid composition. Indeed, the plasma membrane is known to be organized into microdomains, functional units that regulate biological processes associated with the plasma membrane. Often called “lipid rafts”, they are enriched in sphingolipids, sterols, and phospholipids with essentially saturated fatty acids (Bessueille et al. 2009). Proteomic analyses have already revealed the presence of carbohydrate-synthesizing proteins in detergent-resistant domains, suggesting that callose and cellulose synthesis occur in membrane domains with a specific lipid composition (Briolay et al. 2009; Bessueille et al. 2009). In the pollen tube, the asymmetric distribution of proteins and lipids in the plasma membrane is a fundamental requirement for tip growth. The pollen tube apex is characterized by growth-related events, including calcium ion influx, ROS production, secretory vesicle fusion, and GTPase activation/deactivation (Potocký et al. 2012). In this work, we found that CalS is specifically associated with detergent-resistant domains in the plasma membrane of the pollen tube. This indicates that, once in the plasma membrane, CalS compartmentalizes in association with specific lipids required for its function (Brownfield et al. 2007, 2008). There is no conclusive evidence for the role of microtubules in establishing detergent-resistant microdomains in pollen tubes (as well as in plant cells) (McKenna et al. 2014). However, it is intriguing to hypothesize that a complex of microtubules, CalS, and lipids may delimit CalS diffusion or activate it to allow callose synthesis in specific areas of the plasma membrane. Indeed, callose forms a tubular sheath surrounding the pollen tube (except at the apex), and the occasional presence of callose at the tube apex is a marker of growth cessation. It is concluded that the regulation of callose distribution is very critical, and the above hypothesis could explain the characteristics of CalS and its distribution along the pollen tube.

## Supplementary Information

Below is the link to the electronic supplementary material.

Supplementary file1 Supplementary Figure 1. Images of actual blots obtained after native electrophoresis. Please refer to Figure 3. (TIF 428 KB)

## Data Availability

The datasets generated during and/or analyzed during the current study are available from the corresponding author on reasonable request.
