# Supplementary figures and images for: Biochemical and cytological interactions between callose synthase and microtubules in the tobacco pollen tube
Source: Plant Cell Rep. 2022 Mar 18;41(5):1301–18. doi: 10.1007/s00299-022-02860-3 (PMC9110548; doi:10.1007/s00299-022-02860-3)

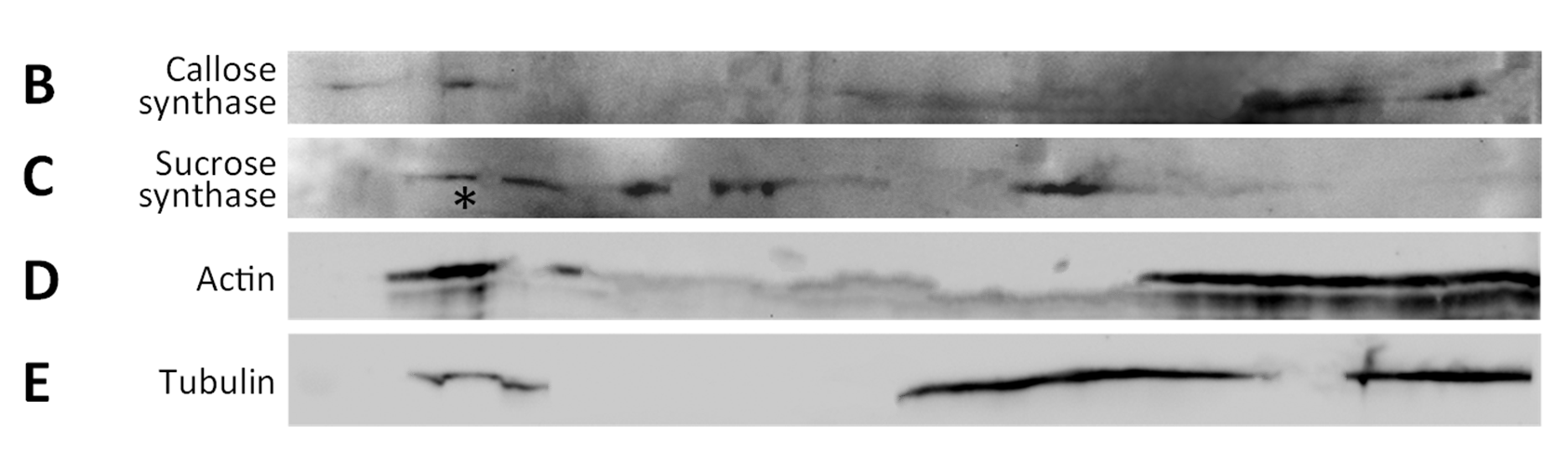

Supplement: Supplementary file 1 — Supplementary file1 Supplementary Figure 1. Images of actual blots obtained after native electrophoresis. Please refer to Figure 3. (TIF 428 KB) [file 299_2022_2860_MOESM1_ESM.tif]
